# Personal

**Published:** 1866-01

**Authors:** 


PERSONAL.—Dr. and Mrs. James P. White, of this city, sailed from New York on Friday, January 5th, in the steamer "Europa" for Brest. Dr. White expects to spend at least eighteen months in Europe, and our readers have the promise of communications upon matters of medical interest observed during his journey in foreign countries. He goes out with the best wishes of his medical friends for a pleasant journey and a safe return.

